# Time-discounting and tobacco smoking: a systematic review and network analysis

**DOI:** 10.1093/ije/dyw233

**Published:** 2016-11-05

**Authors:** Pepita Barlow, Martin McKee, Aaron Reeves, Gauden Galea, David Stuckler

**Affiliations:** 1 Department of Sociology, University of Oxford, Oxford, UK; 2 Department of Public Health and Policy, London School of Hygiene & Tropical Medicine, London, UK; 3 International Inequalities Institute, London School of Economics, London, UK and; 4 Division of Noncommunicable Diseases and Life-course, Regional Office for Europe, World Health OrganizationCopenhagen, Denmark

**Keywords:** time-discounting, time preference, smoking, cessation

## Abstract

**Background:** Tobacco smoking harms health, so why do people smoke and fail to quit? An explanation originating in behavioural economics suggests a role for time-discounting, which describes how the value of a reward, such as better health, decreases with delay to its receipt. A large number of studies test the relationship of time-discounting with tobacco outcomes but the temporal pattern of this relationship and its variation according to measurement methods remain unclear. We review the association between time-discounting and smoking across (i) the life course, from initiation to cessation, and (ii) diverse discount measures.

**Methods:** We identified 69 relevant studies in Web of Science and PubMed. We synthesized findings across methodologies and evaluated discount measures, study quality and cross-disciplinary fertilization.

**Results:** In 44 out of 54 studies, smokers more greatly discounted the future than non-smokers and, in longitudinal studies, higher discounting predicted future smoking. Smokers with lower time-discount rates achieved higher quit rates. Findings were consistent across studies measuring discount rates using hypothetical monetary or cigarette reward scenarios. The methodological quality of the majority of studies was rated as ‘moderate’ and co-citation analysis revealed an isolation of economics journals and a dearth of studies in public health.

**Conclusion:** There is moderate yet consistent evidence that high time-discounting is a risk factor for smoking and unsuccessful cessation. Policy scenarios assuming a flat rate of population discounting may inadequately capture smokers’ perceptions of costs and benefits.

## Introduction

Tobacco smoking is a risk factor for a number of chronic diseases including cancer, lung diseases and cardiovascular diseases.^1^ Many policy-makers have committed vast sums of money to help smokers quit, have increased taxes on cigarettes to raise prices and have mandated graphic labels on cigarette packages to inform smokers of the grave dangers to health.^2^ Ultimately, however policy-makers attempt to reduce the prevalence of smoking, they influence people’s behaviour. Thus, a critical question in public health is: ‘Why do people engage in risky behaviours such as smoking despite evidence and knowledge of their consequences?’

Key Messages

- A potential reason why people smoke or respond poorly to cessation treatments is that they devalue, or ‘discount’, future rewards more than non-smokers and those who successfully quit.
- A large number of studies have tested this hypothesis but there is substantial variation in studies’ methodologies and temporal patterns being analysed. We review the association between time-discounting and smoking across diverse methodologies, including studies of initiation and cessation.
- We find consistent yet moderate quality evidence that smokers more greatly discount the future than non-smokers. Higher discounting predicted future smoking and decreased cessation success; these findings were most consistent with discount measures based on hypothetical monetary or cigarette reward scenarios.
- Co-citation analysis revealed an isolation of economics journals and a dearth of studies in public health.
- Policy scenarios assuming a flat rate of population discounting may inadequately capture smokers’ perceptions of costs and benefits.

One cognitive factor receiving increasing attention in risky behaviours such as tobacco use is the role of time-discounting. Time-discounting characterizes how individuals’ preference for a reward decreases with the delay to its receipt.^3^ Most people tend to prefer smaller, immediate rewards to larger ones available after a delay—i.e. they ‘discount’ the value of future rewards.^4^ Importantly, time-discounting differs from ‘time preference’, which describes whether and how people consider events in the past, present and future when making decisions.^5^ Instead, time-discounting captures the degree to which people devalue rewards with every additional unit of delay—a parameter known as the ‘discount rate’. There is robust evidence that discount rates vary according to multiple socio-economic characteristics and behaviours. For example, discount rates are found to be lower in older and more educated individuals with higher socio-economic status, whilst those who save less for retirement, who gamble and who are overweight have higher discount rates.^6–10^

The health costs of cigarette smoking come at a delay whilst benefits are immediate. Thus, time-discounting may act as one important mediating, and potentially modifiable, factor linking environmental, social and life-course factors to risky unhealthy behaviours, including smoking.^8^ Over the past two decades, a growing body of scholarship has therefore begun testing whether time-discounting correlates with a range of smoking-related behaviours. Studies in this field analyse a range of temporal patterns, including the relationship of time-discounting with smoking initiation, smoking status and dependence at a fixed point in time and sustained cessation.^13–15^ Studies also employ a wide range of alternative discount measures, reflecting differences in research questions, data collection and data analysis across psychological and social sciences.^16^

Understanding the temporal relationship of time-discounting with smoking has important implications for epidemiologists by identifying a potential upstream determinant of persistent tobacco use, mortality and inequalities, and by providing insights that increase the effectiveness of cessation or upstream prevention interventions. Since time-discounting is also correlated with alcohol consumption and poor diet, modifying discount rates to prevent smoking can also provide possible ‘spillover’ benefits by reducing the risk of multiple risky behaviours and associated diseases.^17^^,^^18^

Previous systematic reviews importantly helped to reveal the significance of time-discounting as a cognitive risk factor for smoking. These include a meta-analysis by MacKillop *et al.* that identified higher discount rates among smokers in 79% of the included studies, although the authors exclude studies of initiation and cessation and studies using non-monetary discount rate measures (*n* = 19 studies).^9^ These findings are consistent with a second review by Story *et al.* who also found that higher discount rates for money are associated with several unhealthy behaviours, including smoking (*n* = 39 studies), but excluded studies in social science.^4^ Hughes *et al.* explore possible bi-directionality of the relationship between smoking and discounting, reporting mixed results in experimental studies that test whether elevated time-discounting is a symptom of tobacco withdrawal (*n* = 6 studies).^19^

Previous reviews were relatively narrow in scope, as they do not synthesize temporal patterns of initiation and cessation and heterogeneity according to measurement methods. As MacKillop *et al.*^9^ concluded, there is a ‘need for a better understanding of the chronological relationship’ of time-discounting with smoking (p. 316). To address these gaps in the literature, our review analyses the relationship between smoking and time-discounting across the life course for the first time, from initiation through to cessation. We disaggregate studies by design and methodology to study variation in study findings according to discount rate measurement. We also analyse co-citation patterns to assess whether studies of time-discounting are currently being acknowledged in epidemiology and public health or are instead located in disciplinary siloes. As is being increasingly noted elsewhere, including a recent article in *IJE*, this is especially important in epidemiology and public health, as a failure to include work from other disciplines could lead to partial or incorrect conclusions.^10^^–^^13^ This also enables us to identify opportunities for future research in this cross-disciplinary area of epidemiology and behavioural economics.

## Data and methods

### Search strategy and study selection

We searched all journal fields (title, abstract, subject and full text) in Web of Science and PubMed using the terms described in Table 1. Searching across both databases enabled us to include studies published in journals from a range of disciplines, including economics, pharmacology and neuroscience. Both databases provide journal citation data that can be used for co-citation analysis.

**Table 1.** Search terms

‘time preference’ smoking
‘time preferences’ smoking
‘time-preference’ smoking
‘time-preferences’ smoking
delay discount smoking
delay discounting smoking
delay-discount smoking
delay-discounting smoking
discount rate smoking
inter temporal smoking
inter-temporal smoking
intertemporal smoking
time discount smoking
time discounting smoking
time-discount smoking
time-discounting smoking
‘time preference’ tobacco
‘time preferences’ tobacco
‘time-preference’ tobacco
delay discount tobacco
delay discounting tobacco
delay-discount tobacco
delay-discounting tobacco
discount rate tobacco
inter temporal tobacco
inter-temporal tobacco
intertemporal tobacco
time discount tobacco
time discounting tobacco
time-discount tobacco
time-discounting tobacco

**Figure 1.**
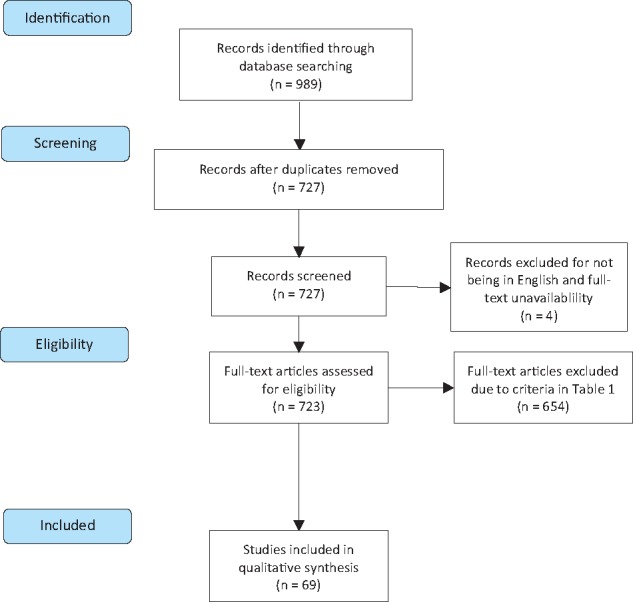
PRISMA flow diagram. *Notes*: PRISMA flow diagram following Moher et al. 2009. Please see Web Appendix 1 for full PRISMA statement.

Figure 1 shows a PRISMA flow diagram depicting study identification, screening and exclusion (see Supplementary Appendix 1, available as Supplementary data at *IJE* online, for full PRISMA statement).^20^ The search yielded 727 unique articles, ranging from 1977 to mid-2015. Papers were excluded if they were (i) not in English, (ii) had been retracted or (iii) were not journal articles or were not yet published. We further excluded those that were not relevant to the study’s objectives, using criteria shown in Table 2. Briefly, the most common reasons were that they failed to study tobacco smoking (378 studies); failed to measure a time-discount rate (125 studies); or they alternatively performed economic cost–benefit analyses (70 studies). Next, we reviewed the bibliographies of previous reviews and book chapters on time-discounting and smoking, although this did not yield any additional studies. Screening and exclusion were conducted by the lead author (PB). Our final analytical sample included 69 studies, covering years 1990 to 2015.

**Table 2.** Study exclusion

Analyses economic costs and benefits
Studies animal subjects (e.g. rats)
Not an empirical study (e.g. systematic review, book chapter, commentary)
Does not measure time discount rates
Article unavailable (e.g. poster or conference abstract, has been retracted, not yet published)
Cannabis not tobacco smoking
Not in English
Outcome variable not smoking initiation, smoking cessation, smoking abstinence, smoking status or quantity of cigarettes consumed per day

### Data extraction and analysis

We extracted title, author, journal, abstract and year for each paper, as well as research question, methodology, sample size, sample demographic, discounting measure (e.g. real or hypothetical rewards, hyperbolic or exponential discounting), smoking variables (e.g. smoker status, dependency, abstinence, cessation success) and main results (see Supplementary Appendix 2, available as Supplementary data at *IJE* online, for study coding). We analysed study results by performing a qualitative synthesis rather than a meta-analysis in order to explore heterogeneity across different methodologies.

We then used an adapted version of the Quality Assessment Tool for Quantitative Studies (developed by the Effective Public Health Practice Project)^21^ to assess the methodological quality of the included studies (Supplementary Appendix 3, available as Supplementary data at *IJE* online). Studies can have between six and eight component ratings, with each component score ranging from 1 (low risk of bias; high methodological quality) to 3 (high risk of bias; low methodological quality). An overall rating for each study was determined based on the component ratings.

Co-citation data were collected from Web of Science and PubMed and analysed using VOSviewer version 1.6.1 and network clustering algorithms.^22^ Sixty-five journals were included based on having at least 10 citations.

## Results

First, we describe trends and disciplinary origins of the reviewed literature. Next, we review the findings from our methodological quality assessment, analyse the main findings by study question and review the different methods used to measure discount rates.

### Trends in and types of publications on time-discounting and smoking

**Figure 2.**
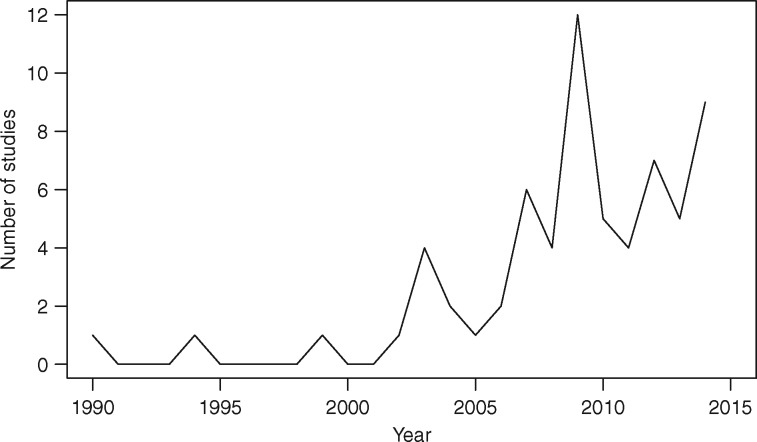
Number of time-discounting and smoking studies published per year, 1990-2014. *Notes*: graph shows the number of studies published per year that were not excluded based on the screening and exclusion criteria in Table 2. Studies from 2015 not included as search was conducted part way through the year.

Figure 2 plots the annual number of studies included in our analysis. There was a marked increase in the number published each year after 2003, when the majority (88.4%) were published.

Among the 69 articles included in our review, 54 were cross-sectional studies of smoking prevalence, 11 were studies of cessation and relapse, and 4 studied initiation and the life course. Sample sizes ranged from 30 to 42,863, with a mean of 1,171, albeit right skewed (median *n* = 80). Sixty-two out of the 69 included studies were conducted in the USA and 7 studies were conducted in Japan.

Table 3 describes the results of the quality assessment of these papers. Most studies were ‘weak’ in representing the general population, tending to rely on college students. However, data collection and reporting quality tended to be high.

**Table 3.** Quality assessment scores

Criterion	Strong	Moderate	Weak
Study design	1	68	0
Confounders	5	63	1
Blinding	0	69	0
Data collection	51	16	2
Representativeness I	1	3	64
Representativeness II	4	10	3
Data analysis	69	0	0
Reporting	45	22	2
Overall rating	1	65	3

Notes: Total studies for representativeness scores do not sum to 69 as 1 article was not eligible for scoring on ‘Representativeness I' and 52 articles were not eligible for scoring on ‘Representativeness II'.

### Time-discounting and smoking initiation

We first evaluated studies looking at initiation, principally occurring among youth. Three longitudinal studies found high discounting corresponded to greater likelihood of initiation. Kang and Ikeda, using the Japanese Household Panel Survey on Consumer Preferences and Satisfaction (*n* = 10 638), found that each standard deviation increase in discount rates was associated with a 4.5 [95% confidence interval (CI): 4.1–4.7] percentage point higher probability of becoming a smoker as well as greater daily cigarette consumption of 1.5 per day (95% CI: 1.22–1.67).^23^ Anokhin *et al.*^24^ used a longitudinal twin design, drawing on repeated interviews with individuals at ages 12 and 14 across three birth cohorts. The authors reported an association of greater preference for immediate rewards with smoking at age 14 (χ^2^ = 8.76, *p* = 0.003) but not at age 12, independently of genetic factors.^24^ Audrain-McGovern *et al.*, using a prospective longitudinal cohort study (*n* = 947) similarly reported that each standard deviation increase in delay discounting increased the odds of smoking initiation by 11% [odds ratio (OR): 1.11, 95% CI: 1.03, 1.23] in youths aged 15–21.^25^

Two retrospective cross-sectional studies found that time-discounting was linked to initiating smoking at younger ages^26^ and smokers’ children had higher discount rates than did non-smokers’.^27^ A cross-sectional study by Reynolds and Fields reported that adolescents experimenting with smoking reported higher discount rates than non-smokers.^28^

### Time-discounting and smoking prevalence

Next, we investigated current smoking behaviour, including 54 cross-sectional studies. Forty-four out of 54 found evidence that discounting increases the likelihood of being a smoker and consuming cigarettes more frequently and in greater quantities.^13^^,^^14^^,^^26–56^ Several studies identified potential modifying characteristics. Seven studies reported results that varied according to gender and cigarette consumption levels, with stronger associations of time-discounting with smoking among men and more dependent smokers.^57–63^ Seven studies found that people with other substance use or mental health problems had stronger associations of smoking and time-discounting, including cocaine-dependent smokers,^64^ those with depressive symptoms,^65^ heavy drinkers,^66^ obese persons^67^ and individuals with gambling problems,^68^ compared with smokers without these symptoms.^69–73^

### Time-discounting and smoking cessation

We then investigated the link of quitting success with time-discounting. Three longitudinal studies report that high time discounters were less successful in cessation. Ida *et al.* found that a 1% increase in the discount parameter at baseline resulted in a 10% increase in the chance of smoking relapse after a 5-month follow-up.^74^ Similarly, Goto *et al.* reported a hazard ratio for relapse of 1.17 (95% CI: 1.10–1.24).^72^ Yoon *et al.* found that baseline time-discounting predicted smoking relapse at 24 weeks post partum among a sample of women who spontaneously quit smoking after discovering they were pregnant.^75^

We also looked at whether discounting modified the effectiveness of cessation interventions. Six studies found that higher time-discounting was linked to less successful abstinence in a cessation intervention, including nicotine replacement therapy, cognitive behavioural therapy and abstinence-contingent monetary rewards.^26^^,^^53^^,^^61–63^ For example, Krishnan-Sarin *et al.*^13^ found that, following a 4-week, high-school-based cessation programme in which participants received weekly monetary rewards if they abstained, individuals with higher discount rates at treatment onset were less likely to have remained abstinent from smoking at the end of the 4-week programme (*F* = 2.67, *p* < 0.05). Two studies, however, found no impact of discounting on the effectiveness of cessation interventions.^76^^,^^77^

### Heterogeneity by discipline and discount measurement

**Figure 3.**
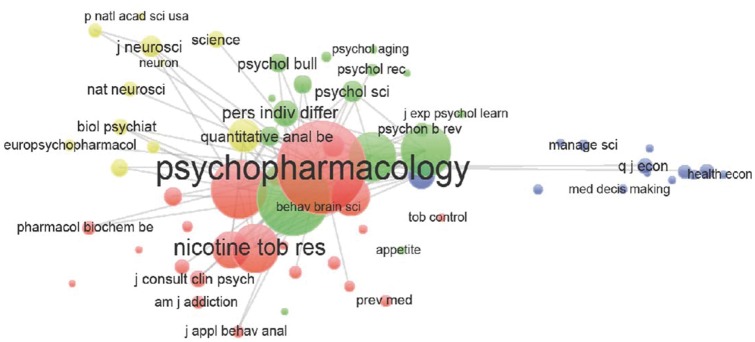
Co-citation of journals. *Notes*: Bubble sizes correspond to the relative magnitude of each journal’s citations in other journals (minimum 10 citations per journal; n = 65 journals). Proximity of bubbles corresponds to the frequency with which journals are cited together in other journals. Colours reflect communities identified by VOS clustering. Produced in VOSviewer Version 1.6.1.

Figure 3 shows journal co-citation patterns revealing four main journal clusters: economics, neuroscience, psychology and pharmacology disciplines. The most highly cited journal is *Psychopharmacology*. The psychology and pharmacology journals have the strongest tendency for co-citation, the neuroscience cluster has a slightly weaker tendency for co-citation and the economics cluster is the most isolated.

Supplementary Appendix 3 (available as Supplementary data at *IJE* online) describes our review of heterogeneity in study findings according to the method used to measure time-discounting. The main methodological differences hinged on whether surveys asked respondents to value hypothetical or potentially real rewards. Studies measuring discount rates using hypothetical money or cigarette rewards tended to find more consistent results, reporting positive associations of time-discounting with adverse smoking behaviour in at least 80% of studies. Results with real or potentially real monetary rewards were less consistent.

## Discussion

Our systematic review highlights several important findings on the relationship between smoking and time-discounting. First, cross-sectional studies consistently report a significantly greater likelihood of smoking, as well as greater quantity and frequency of consumption, among those with high time-discount rates. The association with smoker status was consistent across hypothetical discount rate measures. Second, the small number of longitudinal studies consistently reported a positive association between time-discounting and the likelihood of initiating smoking. Third, smokers with lower time-discount rates achieved higher quit rates and prolonged abstinence with smoking cessation treatment. Fourth, the methodological quality of studies in time-discounting and tobacco smoking was predominantly ‘moderate’, largely due to a strong reliance on convenience sampling and the small number of longitudinal studies. Fifth, co-citation analysis revealed an isolation of economics journals and a dearth of studies in epidemiology and public health.

Our review has several limitations. Due to methodological variation between studies, it was not possible to perform a meta-analysis or to calculate pooled effect sizes. To address this limitation, we have structured the review according to the type of smoking behaviour being analysed, permitting comparisons at different stages of smoking from initiation to cessation. This systematic review also points to several limitations in existing research. First, the bulk of studies is cross-sectional, precluding ascertainment of a clear causal link between smoking and discounting. There is potential bi-directionality in cross-sectional studies, where nicotine exposure increases time-discounting rather than vice-versa, e.g. by inhibiting cortical regulation mechanisms or by impacting the sensitivity of the brain’s reward system.^52^^,^^78^ Previous studies not included in our review found that (i) those seeking smoking cessation had lower discount rates, creating potential selection bias that is unaddressed in experiments analysing voluntary participants,^25^ and (ii) time-discounting significantly decreased at a 12-month follow-up among smokers who successfully quit.^79^ Conversely, there is also evidence that short-term withdrawal from smoking increases time-discounting.^80^ This suggests a potentially complex aetiology, where time-discounting is a risk factor for smoking, yet abstinence increases time-discounting in the short run but causes a reduction in discounting in the long run. Future research should address these limitations and clarify the causal link between smoking and discounting.

Second, there was also a strong reliance on convenience sampling, limiting external validity and leading to a ‘moderate’ quality rating for the majority of studies in this field. Most studies were also conducted in the USA and Japan. There is variation in time-discounting and its association with smoking between different age groups,^24^ suggesting that a reliance on local student populations comprising young adults is a limit to external validity.

Third, as revealed by co-citation analysis, there is a dearth of studies in epidemiology and public health. Additionally, the field of economics is relatively isolated. This suggests that the findings identified in our review are not currently acknowledged in public health and in epidemiology despite their important implications. For example, it is possible that epidemiological studies which do not account for the potential contribution of time-discounting to multiple behaviours may attribute an observed health outcome to smoking rather than other risky behaviours such as diet, physical inactivity and excess alcohol consumption.

Few studies analysed the life-course origins of discount rates in order to test whether these explain the social patterning of smoking.^27^ Studies in sociology and social psychology indicate the importance of social and environmental factors for both smoking and discounting, e.g. via their effects on cognitive function. Poor cognitive function is linked to higher discounting and is hampered by stress, leading Bickel and colleagues, in a recent review, to highlight the potential role of stress-related socio-economic factors such as poverty in influencing unhealthy behaviour via increased discounting.^8^ Research into the life-course determinants of cognitive function highlights the additional roles of language development, home learning environments, parenting style and beliefs, and health (maternal health, birth weight and breastfeeding).^81–83^ The social patterning of these environmental factors corresponds to social gradients in smoking, suggesting that all could contribute to smoking behaviour via their knock-on effects on time-discounting. Whilst all longitudinal studies controlled for a small number of socio-demographic characteristics, adjustment for multiple socio-economic characteristics was less common. Three of the six longitudinal studies adjusted for education, one study controlled for employment status and one study adjusted for income. There is therefore possible omitted variable bias across the majority of longitudinal studies, with just one out of six studies adjusting simultaneously for age, education and employment status.^75^ In our review, one study also identified different associations of time-discounting with smoking at different ages.^24^ Thus, there may exist a ‘critical juncture’ in the life course, at which point time-discounting emerges as a risk factor for smoking. Future research should address this limitation in the literature by identifying the social and life-course determinants of time-discounting. This will be important to identify policy implications for potential upstream rather than technical interventions that mitigate the risk of smoking, whilst also identifying the social groups for whom cessation interventions may be least effective.

Our review identified that the cross-sectional association between time-discounting and smoker status was consistent across studies using hypothetical monetary and cigarette measurement methods. Previous studies identify a correlation between discount rates measured using hypothetical monetary rewards and measures using real and appetitive rewards including cigarettes, although discount rate estimates tended to be smaller for hypothetical rewards.^16^ Thus, discount rate estimates using hypothetical cigarette or monetary rewards may underestimate discount rates for real cigarettes. Yet, our review identified a consistent correlation with smoker status across hypothetical cigarette and monetary rewards. There is therefore evidence of measurement specificity concerning the scale of discount rates but not the correlation with smoker status. This suggests that hypothetical measures are suitable for future research on time-discounting and tobacco use.

There is nevertheless a need for further research to test the construct validity of currently utilized discount rate measures. The majority of existing studies focus on discount rates for one reward at a time (cigarettes, money, health) whilst the theoretical mechanisms underlying the link with smoking behaviour involves a trade-off between two different rewards: cigarettes and health. There is also potential confounding in assessing time-discounting with risk-aversion if delayed rewards are viewed as more uncertain^50^ yet a small proportion of studies accounted for this possibility. Further, insights from behavioural economics also suggest that gains are discounted more steeply than losses.^84–86^ There is a need for future research to better identify the specific types of discounting (risk vs delay, gains vs losses, cigarettes vs money) that increase risks of smoking, increase during abstinence and decrease the likelihood of successful cessation.

Despite these limitations, our review identified evidence that high time-discounting is a risk factor for smoking, including initiation and unsuccessful cessation. This has important implications for tobacco interventions. Although tobacco-related mortality and initiation have begun to decline in many countries, there are still important challenges that need to be addressed. Inequalities in tobacco initiation and mortality remain stable in many countries.^87^ Tobacco use continues to rise in some contexts, particularly low- and middle-income countries.^88^ Cessation programmes have not always been effective, benefitting some groups more than others.^89^^,^^90^ The findings from our review show that time-discounting can potentially help epidemiologists, policy-makers and public health practitioners understand these trends and develop interventions that can tackle such issues. First, interventions which assume a flat rate of discounting across population groups may fail to capture smokers’ perception of costs and benefits over time. There appears to be a critical window during initial withdrawal when discount rates may increase. Second, higher discount rates may be a common cognitive risk factor, as a so-called ‘trans-disease process’, playing a role in several unhealthy behaviours including hazardous alcohol consumption and binge eating.^91^ There may be scope to identify policies and interventions that influence discount rates, with a specific focus on elevated rates during initial withdrawal, as a useful target for intervention that simultaneously reduces the risks of multiple chronic non-communicable diseases.^18^ For example, Radu *et al.* find that discount rates can be reduced by re-framing intertemporal choices from ‘nothing now but more later’ to ‘something now but nothing later’.^92^ In addition, working memory training has been found to decrease the degree to which individuals discount delayed monetary rewards.^93^ Interventions that address differences in time-discounting cannot replace existing interventions but they may offer a complementary approach that can inform how other interventions are communicated to reduce initiation, increase cessation and minimize inequalities.

## Supplementary Data

Supplementary data are available at *IJE* online.

## Funding

PB was funded by WHO Regional Office for Europe. DS was funded by the Wellcome Trust and ERC HRES 313590.

## Supplementary Material

Supplementary Web Appendix 1-4

Supplementary Web Appendix Summaries

## References

[1] WHO. WHO Global Report on Trends in Prevalence of Tobacco Smoking. WHO, 2015.

[2] Britton J, Bogdanovica I. Tobacco control efforts in Europe. The Lancet 2013; **381**:1588–95. 10.1016/S0140-6736(13)60814-4 23642700

[3] Myerson J, Green L, Warusawitharana M. Area under the curve as a measure of discounting. J Exp Anal Behav 2001;76:235–43. 11599641 10.1901/jeab.2001.76-235 PMC1284836

[4] Story GW, Vlaev I, Seymour B, Darzi A, Dolan RJ. Does temporal discounting explain unhealthy behavior? A systematic review and reinforcement learning perspective. Front Behav Neurosci 2014;8:76. 24659960 10.3389/fnbeh.2014.00076 PMC3950931

[5] Adams J. Time for a change of perspective on behavior change interventions? Addiction 2009;104:1025–6. 19466927 10.1111/j.1360-0443.2009.02620.x

[6] Read D, Read NL. Time discounting over the lifespan. Organ Behav Hum Decis Process 2004;94:22–32.

[7] Frederick S, Loewenstein G, Donoghue TO, Donoghue TEDO. Time discounting and preference: a critical review. J Econ Lit 2002;40:351–401.

[8] Bickel W, Moody L, Quisenberry AJ, Ramey CT, Sheffer CE. A competing neurobehavioral decision systems model of SES-related health and behavioral disparities. Prev Med (Baltim) 2014;68:37–43. 10.1016/j.ypmed.2014.06.032 PMC4253853 25008219

[9] MacKillop J, Amlung MT, Few LR, Ray LA, Sweet LH, Munafò MR. Delayed reward discounting and addictive behavior: a meta-analysis. Psychopharmacology (Berl) 2011;216:305–21. 21373791 10.1007/s00213-011-2229-0 PMC3201846

[10] Barlow P, Reeves A, McKee M, Galea G, Stuckler D. Unhealthy diets, obesity and time discounting: a systematic literature review and network analysis. Obes Rev 2016;7:1–10. 10.1111/obr.12431 PMC4988386 27256685

[11] Stuckler D, Basu S, Suhrcke M, McKee M. The health implications of financial crisis: a review of the evidence. *Ulster Med J* 2009;**78**:142–5. PMC2773609 19907678

[12] Trinquart L, Johns DM, Galea S. Why do we think we know what we know? A metaknowledge analysis of the salt controversy. *Int J Epidemiol* 2016;**45**(1):251–60. 10.1093/ije/dyv184 26888870

[13] Sato M, Ohkusa Y. The relationship between smoking initiation and time discount factor, risk aversion and information. Applied Economics Letters 2003; **10**:287–9.

[14] Bickel WK, Odum AL, Madden GJ. Impulsivity and cigarette smoking: delay discounting in current, never, and ex-smokers. Psychopharmacology (Berl) 1999;146:447–54. 10550495 10.1007/pl00005490

[15] Krishnan-Sarin S, Reynolds B, Duhig AM Behavioral impulsivity predicts treatment outcome in a smoking cessation program for adolescent smokers. Drug Alcohol Depend 2007;88:79–82. 17049754 10.1016/j.drugalcdep.2006.09.006 PMC1868698

[16] Odum AL, Baumann AAL. Cigarette smokers show steeper discounting of both food and cigarettes than money. Drug and Alcohol Dependence 2007; **91**:293–6. 17720334 10.1016/j.drugalcdep.2007.07.004

[17] Bickel WK, Jarmolowicz DP, Mueller ET, Koffarnus MN, Gatchalian KM. Excessive discounting of delayed reinforcers as a trans-disease process contributing to addiction and other disease-related vulnerabilities: emerging evidence. *Pharmacology and* Therapeutics 2012; **134**:287–97. 10.1016/j.pharmthera.2012.02.004 PMC3329584 22387232

[18] Bickel WK, MacKillop J, Madden GJ, Odum AL, Yi R. Experimental manipulations of delay discounting & related processes: an introduction to the special issue. J Exp Anal Behav 2015;103:1–9. 25641079 10.1002/jeab.133 PMC5523657

[19] Hughes JR, Dash M, Callas PW. Is impulsivity a symptom of initial tobacco withdrawal? A meta-analysis and qualitative systematic review. Nicotine Tob Res 2014; **17**:503–9. 25335950 10.1093/ntr/ntu220

[20] Moher D, Liberati A, Tetzlaff J, Altman DG, The PRISMA Group. Preferred reporting items for systematic reviews and meta-analyses: the PRISMA statement. Ann Intern Med. 2009;151:264–9. 19622511 10.7326/0003-4819-151-4-200908180-00135

[21] Thomas BH, Ciliska D, Dobbins M, Micucci S. A process for systematically reviewing the literature: providing the research evidence for public health nursing interventions. Worldviews Evidence-Based Nurs 2004;1:176–84. 10.1111/j.1524-475X.2004.04006.x 17163895

[22] van Eck NJ, Waltman L. Software survey: VOSviewer, a computer program for bibliometric mapping. Scientometrics 2010;84:523–38. 20585380 10.1007/s11192-009-0146-3 PMC2883932

[23] Kang M-I, Ikeda S. Time discounting and smoking behaviour: evidence from a panel survey. Health Econ 2014;23:1443–64. 24136867 10.1002/hec.2998

[24] Anokhin AP, Golosheykin S, Grant JD, Heath AC. Heritability of delay discounting in adolescence: a longitudinal twin study. Behav Genet 2011;41:175–83. 20700643 10.1007/s10519-010-9384-7 PMC3036802

[25] Audrain-McGovern J, Rodriguez D, Epstein LH, Cuevas J, Rodgers K, Wileyto EP. Does delay discounting play an etiological role in smoking or is it a consequence of smoking? Drug Alcohol Depend 2009;103:99–106. 19443136 10.1016/j.drugalcdep.2008.12.019 PMC2743449

[26] Kollins SH. Delay discounting is associated with substance use in college students. Addict Behav 2003;28:1167–73. 12834659 10.1016/s0306-4603(02)00220-4

[27] Reynolds B, Leraas K, Collins C, Melanko S. Delay discounting by the children of smokers and nonsmokers. Drug Alcohol Depend 2009;99:350–3. 18818028 10.1016/j.drugalcdep.2008.07.015

[28] Reynolds B, Fields S. Delay discounting by adolescents experimenting with cigarette smoking. Addiction 2012;107:417–24. 21906199 10.1111/j.1360-0443.2011.03644.x PMC3260343

[29] Odum AL, Madden GJ, Bickel WK. Discounting of delayed health gains and losses by current, never- and ex-smokers of cigarettes. Nicotine Tob Res 2002;4:295–303. 12215238 10.1080/14622200210141257

[30] Reynolds B. The experiential discounting task is sensitive to cigarette-smoking status and correlates with a measure of delay discounting. Behavioural Pharmacology 2006; 133–42. 10.1097/01.fbp.0000190684.77360.c0 16495721

[31] Reynolds B, Richards JB, Horn K, Karraker K. Delay discounting and probability discounting as related to cigarette smoking status in adults. Behav Processes 2004;65:35–42. 14744545 10.1016/s0376-6357(03)00109-8

[32] Mitchell SH, Wilson VB. Differences in delay discounting between smokers and nonsmokers remain when both rewards are delayed. Psychopharmacology (Berl) 2012;219:549–62. 21983917 10.1007/s00213-011-2521-z PMC3677053

[33] Lawyer SR, Schoepflin F, Green R, Jenks C. Discounting of hypothetical and potentially real outcomes in nicotine-dependent and nondependent samples. Exp Clin Psychopharmacol 2011;19:263–74. 21707190 10.1037/a0024141

[34] Chabris CF, Laibson D, Morris CL, Schuldt JP, Taubinsky D. Individual laboratory-measured discount rates predict field behavior. J Risk Uncertain 2008;37:237–69. 19412359 10.1007/s11166-008-9053-x PMC2676104

[35] Bickel WK, Jarmolowicz DP, Mueller ET, Franck CT, Carrin C, Gatchalian KM. Altruism in time: social temporal discounting differentiates smokers from problem drinkers. Psychopharmacology (Berl) 2012;224:109–20. 22644127 10.1007/s00213-012-2745-6 PMC10449014

[36] Ida T. A quasi-hyperbolic discounting approach to smoking behavior. Health Econ Rev 2014;4:5. 25006542 10.1186/s13561-014-0005-7 PMC4077625

[37] Reimers S, Maylor EA, Stewart N, Chater N. Associations between a one-shot delay discounting measure and age, income, education and real-world impulsive behavior. Pers Individ Dif 2009;47:973–8.

[38] Bickel WK, Yi R, Kowal BP, Gatchalian KM. Cigarette smokers discount past and future rewards symmetrically and more than controls: Is discounting a measure of impulsivity? Drug Alcohol Depend 2008;96:256–62. 18468814 10.1016/j.drugalcdep.2008.03.009 PMC2701143

[39] Sweitzer MM, Donny EC, Dierker LC, Flory JD, Manuck SB. Delay discounting and smoking: association with the Fagerström Test for Nicotine Dependence but not cigarettes smoked per day. Nicotine Tob Res 2008;10:1571–5. 18946776 10.1080/14622200802323274

[40] Baker F, Johnson MW, Bickel WK. Delay discounting in current and never-before cigarette smokers: similarities and differences across commodity, sign, and magnitude. J Abnorm Psychol 2003;112:382–92. 12943017 10.1037/0021-843x.112.3.382

[41] Wing VC, Moss TG, Rabin RA, George TP. Effects of cigarette smoking status on delay discounting in schizophrenia and healthy controls. Addict Behav 2012;37:67–72. 21963152 10.1016/j.addbeh.2011.08.012

[42] Stillwell DJ, Tunney RJ. Effects of measurement methods on the relationship between smoking and delay reward discounting. Addiction 2012;107:1003–12. 22126134 10.1111/j.1360-0443.2011.03742.x

[43] Ida T, Goto R. Interdependency among addictive behaviours and time/risk preferences: discrete choice model analysis of smoking, drinking, and gambling. J Econ Psychol 2009;30:608–21.

[44] Takahashi T, Oono H, Ohmura Y, Kitamura N, Radford M. Relationship between personality scales of impulsiveness and discounting of monetary gains and losses in smokers and never smokers In: Katlin LJ (ed). Men and Addictions: New Research. Nova Science Publishers, 2009.

[45] Rass O, Ahn W-Y, O’Donnell BF. Resting-state EEG, impulsiveness, and personality in daily and nondaily smokers. Clin Neurophysiol. International Federation of Clinical Neurophysiology, in press. 10.1016/j.clinph.2015.05.007 PMC4644505 26051750

[46] Ida T, Goto R. Simultaneous measurement of time and risk preferences: stated preference discrete choice modeling analysis depending on smoking behavior. Int Econ Rev (Philadelphia) 2009;50(4):1169–82.

[47] Bradford WD. The association between individual time preferences and health maintenance habits. Med Decis Making 2010;30:99–112. 19675322 10.1177/0272989X09342276

[48] Friedel JE, DeHart WB, Madden GJ, Odum AL. Impulsivity and cigarette smoking: discounting of monetary and consumable outcomes in current and non-smokers. Psychopharmacology (Berl) 2014;231:4517–26. 24819731 10.1007/s00213-014-3597-z PMC4221621

[49] Fields S, Collins C, Leraas K, Reynolds B. Dimensions of impulsive behavior in adolescent smokers and nonsmokers. Exp Clin Psychopharmacol 2009;17:302–11. 19803629 10.1037/a0017185 PMC3209711

[50] Reynolds B, Patak M, Shroff P. Adolescent smokers rate delayed rewards as less certain than adolescent nonsmokers. Drug Alcohol Depend 2007;90:301–3. 17543476 10.1016/j.drugalcdep.2007.04.008 PMC1991332

[51] Peters J, Bromberg U, Schneider S Lower ventral striatal activation during reward anticipation in adolescent smokers. Am J Psychiatry 2011;168:540–9. 21362742 10.1176/appi.ajp.2010.10071024

[52] Reynolds B. Do high rates of cigarette consumption increase delay discounting? A cross-sectional comparison of adolescent smokers and young-adult smokers and nonsmokers. Behav Processes 2004;67:545–9. 15519004 10.1016/j.beproc.2004.08.006

[53] Romer D, Duckworth AL, Sznitman S, Park S. Can adolescents learn self-control? Delay of gratification in the development of control over risk taking. Prev Sci 2010;11:319–30. 20306298 10.1007/s11121-010-0171-8 PMC2964271

[54] Rezvanfard M, Ekhtiari H, Mokri A, Djavid GE, Kaviani H. Psychological and behavioral traits in smokers and their relationship with nicotine dependence level. Arch Iran Med 2010;13:395–405. 20804306

[55] Jiga-Boy GM, Storey K, Buehner MJ. Smokers discount their drug of abuse in the same way as other consumable rewards. Q J Exp Psychol (Hove) 2013;June:37–41. 23509964 10.1080/17470218.2013.772646

[56] Daugherty JR, Brase GL. Taking time to be healthy: predicting health behaviors with delay discounting and time perspective. Pers Individ Dif. Elsevier Ltd 2010;48:202–7.

[57] Hornik J. Time preference, psychographics, and smoking behavior. J Health Care Mark 1990;10:36–46. 10104012

[58] López-Torrecillas F, Perales JC, Nieto-Ruiz A, Verdejo-García A. Temperament and impulsivity predictors of smoking cessation outcomes. PLoS One 2014;9:e112440. 25474540 10.1371/journal.pone.0112440 PMC4256301

[59] Clewett D, Luo S, Hsu E, Ainslie G, Mather M, Monterosso J. Increased functional coupling between the left fronto-parietal network and anterior insula predicts steeper delay discounting in smokers. Hum Brain Mapp 2014;35:3774–87. 24523255 10.1002/hbm.22436 PMC6869514

[60] Harrison GW, Lau MI, Rutström EE. Individual discount rates and smoking: evidence from a field experiment in Denmark. J Health Econ. Elsevier B.V. 2010;29:708–17. 10.1016/j.jhealeco.2010.06.006 20727602

[61] Johnson MW, Bickel WK, Baker F. Moderate drug use and delay discounting: a comparison of heavy, light, and never smokers. Exp Clin Psychopharmacol 2007;15:187–94. 17469942 10.1037/1064-1297.15.2.187

[62] Jones BA, Landes RD, Yi R, Bickel WK. Temporal horizon: modulation by smoking status and gender. Drug Alcohol Depend 2009;104(Suppl 1): 87–93. 10.1016/j.drugalcdep.2009.04.001 PMC2732767 19446407

[63] Ohmura Y, Takahashi T, Kitamura N. Discounting delayed and probabilistic monetary gains and losses by smokers of cigarettes. Psychopharmacology (Berl) 2005;182:508–15. 16167142 10.1007/s00213-005-0110-8

[64] García-Rodríguez O, Secades-Villa R, Weidberg S, Yoon JH. A systematic assessment of delay discounting in relation to cocaine and nicotine dependence. Behav Processes 2013;99:100–5. 23872502 10.1016/j.beproc.2013.07.007

[65] Imhoff S, Harris M, Weiser J, Reynolds B. Delay discounting by depressed and non-depressed adolescent smokers and non-smokers. Drug Alcohol Depend 2014;135:152–5. 24360649 10.1016/j.drugalcdep.2013.11.014

[66] Moallem NR, Ray LA. Dimensions of impulsivity among heavy drinkers, smokers, and heavy drinking smokers: singular and combined effects. Addict Behav 2012;37:871–4. 22445419 10.1016/j.addbeh.2012.03.002 PMC3741101

[67] Fields SA, Sabet M, Peal A, Reynolds B. Relationship between weight status and delay discounting in a sample of adolescent cigarette smokers. Behav Pharmacol 2011;22:266–8. 21430520 10.1097/FBP.0b013e328345c855 PMC3119921

[68] Andrade LF, Alessi SM, Petry NM. The effects of alcohol problems and smoking on delay discounting in individuals with gambling problems. J Psychoactive Drugs 2013;45:241–8. 24175489 10.1080/02791072.2013.803645 PMC3816387

[69] Melanko S, Leraas K, Collins C, Fields S, Reynolds B. Characteristics of psychopathy in adolescent nonsmokers and smokers: relations to delay discounting and self reported impulsivity. Exp Clin Psychopharmacol 2009;17(4):258–65. 19653791 10.1037/a0016461

[70] White TJ, Redner R, Skelly JM, Higgins ST. Examining educational attainment, pre-pregnancy smoking rate, and delay discounting as predictors of spontaneous quitting among pregnant smokers. Exp Clin Psychopharmacol 2014;22:384–91. 25069014 10.1037/a0037492 PMC4180793

[71] Jaroni JL, Wright SM, Lerman C, Epstein LH. Relationship between education and delay discounting in smokers. Addict Behav 2004;29:1171–5. 15236819 10.1016/j.addbeh.2004.03.014

[72] Goto R, Takahashi Y, Nishimura S, Ida T. A cohort study to examine whether time and risk preference is related to smoking cessation success. *Addiction* 2009;**104**(6):1018–24. 10.1111/j.1360-0443.2009.02585.x 19466926

[73] Wilson AG, Franck CT, Terry Mueller E Predictors of delay discounting among smokers: education level and a Utility Measure of Cigarette Reinforcement Efficacy are better predictors than demographics, smoking characteristics, executive functioning, impulsivity, or time perception. Addict Behav. Elsevier Ltd 2015;45:124–33. 10.1016/j.addbeh.2015.01.027 PMC4376282 25661991

[74] Ida T, Goto R, Takahashi Y, Nishimura S. Can economic-psychological parameters predict successful smoking cessation? J Socio Econ 2011;40:285–95.

[75] Yoon JH, Higgins ST, Heil SH, Sugarbaker RJ, Thomas CS, Badger GJ. Delay discounting predicts postpartum relapse to cigarette smoking among pregnant women. Exp Clin Psychopharmacol 2007;15:176–86. 17469941 10.1037/1064-1297.15.2.186

[76] Lopez A a, Skelly JM, White TJ, Ph D, Stephen T. Does impulsiveness moderate response to financial incentives for smoking cessation among pregnant and newly postpartum. Exp Clin Psychopharmacol 2015;23:97–108. 25730417 10.1037/a0038810 PMC4388785

[77] Harris M, Penfold RB, Hawkins A, Maccombs J, Wallace B, Reynolds B. Dimensions of impulsive behavior and treatment outcomes for adolescent smokers. Exp Clin Psychopharmacol 2014;22:57–64. 24417209 10.1037/a0034403

[78] Anderson KG, Diller JW. Effects of acute and repeated nicotine administration on delay discounting in Lewis and Fischer 344 rats. Behavioural Pharmacology 2010;**21**:754–64. 20944502 10.1097/FBP.0b013e328340a050 PMC3046322

[79] Secades-villa R, Weidberg S, García-rodríguez O, Fernández-hermida JR, Ho J. Decreased delay discounting in former cigarette smokers at one year after treatment. Addict Behav. Elsevier Ltd 2014;39:1087–93. 10.1016/j.addbeh.2014.03.015 24661901

[80] Mitchell SH. Effects of short-term nicotine deprivation on decision-making: delay, uncertainty and effort discounting. Nicotine Tob Res 2004;6:819–28. 15700917 10.1080/14622200412331296002

[81] Melhuish EC, Phan MB, Sylva K, Sammons P, Siraj-Blatchford I, Taggart B. Effects of the home learning environment and preschool center experience upon literacy and numeracy development in early primary school. J Soc Issues 2008;64:95–114.

[82] Duncan GJ, Brooks-Gunn J, Klebanov PK. Economic deprivation and early childhood development. Child Dev 1994;65:296–318. 7516849

[83] Dearden L, Sibieta L, Sylva K. The socio-economic gradient in early child outcomes: evidence from the Millennium Cohort Study. Longit Life Course Stud 2011;2:19–40.

[84] Thaler R. Some empirical evidence on dynamic inconsistency. Econ Lett 1981;8:201–7.

[85] Kahneman D, Knetsch JL, Thaler RH. Anomalies: the endowment effect, loss aversion, and status quo bias. J Econ Perspect 1991;5:193–206.

[86] Kahneman D, Tversky A. Prospect theory: an analysis of decision under risk. Econometrica 1979;47:263–91.

[87] Nagelhout GE, de Korte-de Boer D, Kunst AE Trends in socioeconomic inequalities in smoking prevalence, consumption, initiation, and cessation between 2001 and 2008 in the Netherlands: findings from a national population survey. BMC Public Health 2012;12:303. 22537139 10.1186/1471-2458-12-303 PMC3356226

[88] Bilano V, Gilmour S, Moffiet T Global trends and projections for tobacco use, 1990–2025: an analysis of smoking indicators from the WHO Comprehensive Information Systems for Tobacco Control. Lancet 2015;385:966–76. 25784347 10.1016/S0140-6736(15)60264-1

[89] Bosdriesz JR,, Willemsen MC,, Stronks K, Kunst a. E. Socioeconomic inequalities in smoking cessation in 11 European countries from 1987 to 2012. J Epidemiol Community Heal 2015;**69**:1–7. 10.1136/jech-2014-205171 25841241

[90] Brown T, Platt S, Amos A. Equity impact of European individual-level smoking cessation interventions to reduce smoking in adults: a systematic review. *Eur* J Public Health 2014;24:551–6. 10.1093/eurpub/cku065 24891458

[91] Bickel WK, Jarmolowicz DP, Mueller ET, Koffarnus MN, Gatchalian KM. Excessive discounting of delayed reinforcers as a trans-disease process contributing to addiction and other disease-related vulnerabilities: emerging evidence. Pharmacol Ther 2012;134:287–97. 22387232 10.1016/j.pharmthera.2012.02.004 PMC3329584

[92] Radu PT, Yi R, Bickel WK, Gross JJ, McClure SM. A mechanism for reducing delay discounting by altering temporal attention. J Exp Anal Behav 2011;96:363–85. 22084496 10.1901/jeab.2011.96-363 PMC3213002

[93] Bickel WK, Yi R, Landes RD, Hill PF, Baxter C. Remember the future: working memory training decreases delay discounting among stimulant addicts. Biol Psychiatry 2011;69:260–5. 20965498 10.1016/j.biopsych.2010.08.017 PMC3015021

