# Corrigendum: Time-discounting and tobacco smoking: a systematic review and network analysis

**DOI:** 10.1093/ije/dyx060

**Published:** 2017-04-12

**Authors:** Pepita Barlow, Martin McKee, Aaron Reeves, Gauden Galea, David Stuckler

First published online: 5 November 2016, *Int J Epidemiol*, 2016, doi: https://doi.org/10.1093/ije/dyw233

A funding statement was missing for author David Stuckler. The funding statement should have included “DS is funded by ERC HRES 313590.”

