# Supplementary material for: Time-discounting and tobacco smoking: a systematic review and network analysis
Source: Int J Epidemiol. 2016 Nov 5;46(3):860–9. doi: 10.1093/ije/dyw233 (PMC5637946; doi:10.1093/ije/dyw233)
Supplement: Supplementary Web Appendix 1-4 [file supplementary_web_appendix_dyw233.docx]

**Web Appendix**

Web Appendix 1: PRISMA statement

Web Appendix 2: studies and study coding (attached as a separate excel file)

Web Appendix 3: Quality assessment tool

Web Appendix 4: Review of heterogeneity by discount measurement method

**Web Appendix 1. PRISMA checklist of items to include when reporting a systematic review or meta-analysis developed by Moher et al. 2009 (1)**

| Section/topic | # | Checklist item | Reported on page # |
| --- | --- | --- | --- |
| TITLE | | | |
| Title | 1 | Identify the report as a systematic review, meta-analysis, or both. | 2 |
| ABSTRACT | | | |
| Structured summary | 2 | Provide a structured summary including, as applicable: background; objectives; data sources; study eligibility criteria, participants, and interventions; study appraisal and synthesis methods; results; limitations; conclusions and implications of key findings; systematic review registration number. | 2 |
| INTRODUCTION | | | |
| Rationale | 3 | Describe the rationale for the review in the content of what is already known. | 4-5 |
| Objectives | 4 | Provide an explicit statement of questions being addressed with reference to participants, interventions, comparisons, outcomes, and study design (PICOS). | 5 |
| METHODS | | | |
| Protocol and registration | 5 | Indicate if a review protocol exists, if and where it can be accessed (e.g., Web address), and, if available, provide registration information including registration number. | 6-7 |
| Eligibility criteria | 6 | Specify study characteristics (e.g., PICOS, length of follow-up) and report characteristics (e.g., years considered, language, publication status) used as criteria for eligibility, giving rationale. | 6 |
| Information sources | 7 | Describe all information sources (e.g., databases with dates of coverage, contact with study authors to identify additional studies) in the search and date last searched. | 6 |
| Search | 8 | Present full electronic search strategy for at least one database, including any limits used, such that it could be repeated. | 6 |
| Study selection | 9 | State the process for selecting studies (i.e., screening, eligibility, included in systematic review, and, if applicable, included in the meta-analysis). | 6 |
| Data collection process | 10 | Describe method of data extraction from reports (e.g., piloted forms, independently, in duplicate) and any processes for obtaining and confirming data from investigators. | 7 |
| Data items | 11 | List and define all variables for which data were sought (e.g., PICOS, funding sources) and any assumptions and simplifications made. | 7 |
| Risk of bias in individual studies | 12 | Describe methods used for assessing risk of bias of individual studies (including specification of whether this was done at the study or outcome level), and how this information is to be used in any data synthesis. | 7 |
| Summary measures | 13 | State the principal summary measures (e.g., risk ratio, difference in means). | n/a |
| Synthesis of results | 14 | Describe the methods of handling data and combining results of studies, if done, including measures of consistency (e.g., I2) for each meta-analysis. | 7 |
| Risk of bias across studies | 15 | Specify any assessment of risk of bias that may affect the cumulative evidence (e.g., publication bias, selective reporting within studies). | 7 |
| Additional analyses | 16 | Describe methods of additional analyses (e.g., sensitivity or subgroup analyses, meta-regression), if done, indicating which were pre-specified. | n/a |
| RESULTS | | | |
| Study selection | 17 | Give numbers of studies screened, assessed for eligibility, and included in the review, with reasons for exclusions at each stage, ideally with a flow diagram. | 6 |
| Study characteristics | 18 | For each study, present characteristics for which data were extracted (e.g., study size, PICOS, follow-up period) and provide the citations. | Web Appendix 2 |
| Risk of bias within studies | 19 | Present data on risk of bias of each study and, if available, any outcome-level assessment (see Item 12). | Web Appendix 2 |
| Results of individual studies | 20 | For all outcomes considered (benefits or harms), present, for each study: (a) simple summary data for each intervention group and (b) effect estimates and confidence intervals, ideally with a forest plot. | Web Appendix 2 |
| Synthesis of results | 21 | Present results of each meta-analysis done, including confidence intervals and measures of consistency. | n/a |
| Risk of bias across studies | 22 | Present results of any assessment of risk of bias across studies (see Item 15). | Box 1; Web Appendix 2 |
| Additional analysis | 23 | Give results of additional analyses, if done (e.g., sensitivity or subgroup analyses, meta-regression [see Item 16]). | n/a |
| DISCUSSION | | | |
| Summary of evidence | 24 | Summarize the main findings including the strength of evidence for each main outcome; consider their relevance to key groups (e.g., health care providers, users, and policy makers). | 11-12 |
| Limitations | 25 | Discuss limitations at study and outcome level (e.g., risk of bias), and at review level (e.g., incomplete retrieval of identified research, reporting bias). | 12-14 |
| Conclusions | 26 | Provide a general interpretation of the results in the content of other evidence, and implications for future research. | 15 |
| FUNDING | | | |
| Funding | 27 | Describe sources of funding for the systematic review and other support (e.g., supply of data); role of funders for the systematic review. | 1 |

**Web Appendix 3. Quality assessment tool**

The Quality Assessment Tool developed by the Effective Public Health Practice Project contains eight key domains, including (1) study design; (2) blinding; (3) representativeness in the sense of selection bias; (4) representativeness in the sense of withdrawals and drop-outs; (5) confounders; (6) data collection; (7) data analysis; (8) reporting. Studies can have between six and eight component ratings, with each component score ranging from 1 (low risk-of bias; high methodological quality) to 3 (high risk-of-bias; low methodological quality) (2).

Rating tool

**STUDY DESIGN**

(**Q1**) The study design is:

1. Experimental
   - 1. Individual-randomised
     2. Group-randomised
     3. Non-randomised
2. Observational
   - 1. Cross-sectional
     2. Longitudinal (also natural experiment or pre-post tests)
     3. Case-control
3. Any other method or did not state method (i.e. pre-post test without control group)

(**Q2**) Was this an intervention study?

**Yes** – proceed

**No** – go to question 7

(**Q3**) Is the intervention of interest clearly described?

1. Yes
2. No

(**Q4**) Were (groups of) subjects randomized into intervention groups?

1. Yes
2. No
3. Not applicable

(**Q5**) Was the intervention assignment concealed from participants and care givers until recruitment was completed?

1. Yes
2. No
3. Can’t tell

(**Q6**) Was (were) the intervention or exposure status of participants concealed from the outcome assessors?

1. Yes
2. No
3. Can’t tell

(**Q7**) Were power/sample size calculations conducted?

1. Yes, details of calculation provided
2. Yes, no details provided
3. Not reported or post hoc computation
4. Not applicable (using an existing database and referring to design article*

**Rating study design: Strong:** Q1 is 1

**Moderate:** Q1 is 2

**Weak:** Q1 is 3

**Rating blinding: Strong:** Q5 and Q6 are 1

**Moderate:** Q5 or Q6 is 1**; or** Q5 or Q6 are 3

**Weak:** Q5 and Q6 are 2; **or** Q5 and Q6 are 3

**(no rate is given when study is not an intervention study)**

*• If the study is using data from a large existing database such as HSE, NHANES, BRFSS etc, often the authors refer to the design paper of the original study and no information in the present article is being described about power calculations, validity of tools et.*

**REPRESENTATIVENESS (selection bias)**

(**Q8**) Is the spectrum of individuals selected to participate likely to be representative of the wider population who experience the intervention/exposure/situation?

1. Very likely
2. Somewhat likely
3. Not likely (selected group of users e.g., volunteers)
4. Can´t tell (no information provided)
5. Not applicable (using an existing database and authors refer to design article)

(**Q9**) What percentage of the selected participants agreed to participate?

1. ⁮ ……….%
2. ⁮ Can’t tell
3. ⁮ Not applicable

(**Q10**) Were inclusion/exclusion criteria specified and number of exclusions reported?

1. Criteria and number of exclusions reported
2. Criteria or number of exclusions not reported
3. Criteria and number not reported

**Rating: Strong:** Q8 is 1

**Moderate:** Q8 is 2

**Weak:** Q8 is 3 or 4

**No rating:** Q8 is 5

**REPRESENTATIVENESS (withdrawals and drop-outs)**

(**Q11**) Were withdrawals and drop-outs reported in terms of numbers and reasons per group?

1. Numbers and reasons provided
2. Numbers but no reasons provided
3. Can’t tell (if longitudinal data)
4. Not applicable (if cross-sectional data or if using an existing database and authors refer to design article)

*If Q11 is 1 or 2, proceed to Q12. Otherwise, proceed to Q13.*

(**Q12**) What was the loss to follow-up/percentage completing the study? (If % differs by groups, record the lowest)

1. ⁮ …………..%
2. ⁮ Not provided
3. ⁮ Not applicable

**Rating: Strong:** Q11 is 1

**Moderate:** Q11 is 2

**Weak:** Q11 is 3

**No rating:** Q11 is 4

**CONFOUNDERS**

(**Q13#**) What confounders were the analyses adjusted for?

…………………………………………………………………………………………………...

…………………………………………………………………………………………………...

(**Q13**) Were analyses appropriately adjusted for confounders?

1. For most confounders
2. For some confounders
3. No or can’t tell

*The following are examples of confounders: race, sex, marital status/family, age, SES (income or class), education, health status, pre-intervention score on outcome measure.*

*Considering the study design, were appropriate methods for controlling confounding variables and limiting potential biases used? Confounding can be addressed by appropriate use of randomization, restriction, matching, stratification, or multivariable methods. Sometimes use of a single method may be inadequate. Some biases can be limited by institution of data collection or study procedures that support validity of the study (e.g. training and/or blinding of interviewers or observers, interviewers and observers are different from interventions’ implementers etc). Example: if between-group differences persist after randomization or matching, statistical control should also have been used.*

**Rating: Strong:** Q13 is 1

**Moderate:** Q13 is 2

**Weak:** Q13 is 3

**DATA COLLECTION**

(**Q14**) Were validity, reliability or appropriateness of the data collection tools discussed?

- 1. Both validity and reliability were discussed
  2. a. Validity or reliability were discussed

b. A national dataset was used and authors provided adequate information to find information on validity and reliability

- 1. None of them were discussed

**Rating: Strong:** Q14 is 1

**Moderate:** Q14 is 2

**Weak:** Q14 is 3

**DATA ANALYSIS**

(**Q15**) Were appropriate statistical analyses conducted (including correction for multiple tests where applicable)?

1. a. Statistical methods were described and were appropriate and comprehensive – sophisticated approach

b. Statistical methods were described and were appropriate and comprehensive –simple approach

1. Statistical methods were described and less appropriate
2. No description of statistical methods or inappropriate methods

**Rating: Strong:** Q15 is 1

**Moderate:** Q15 is 2

**Weak:** Q15 is 3

**REPORTING**

(**Q16**) Are the hypothesis/aim/objective of the study clearly described?

1. Yes
2. No

(**Q17**) Are the main outcomes to be measured clearly described?

1. Yes
2. No

(**Q18**) Are the main findings clearly described?

1. Yes
2. No

(**Q19**) Have actual probability values been reported

*(i.e. p=0.345 instead of p>0.05; same goes for t-values, 95%CIs etc)?*

1. Yes
2. No

**Rating: Strong:** Q16 and Q19 are 1

**Moderate:** Q16 or Q19 are 1

**Weak:** Q16 and Q19 are 2

Studies can have between six and eight component ratings. The overall rating for each study is determined by assessing the component ratings.

If seven ratings have been given;

**Strong** will be attributed to those with no WEAK ratings and at least four STRONG ratings;

**Moderate** will be given to those with one WEAK rating or fewer than four STRONG ratings;

**Weak** will be attributed to those with two or more WEAK ratings.

If six ratings have been given;

**Strong** will be attributed to those with no WEAK ratings and at least three STRONG ratings; **Moderate** will be given to those with one WEAK rating or fewer than three STRONG ratings;

**Weak** will be attributed to those with two or more WEAK ratings.

If five ratings have been given;

**Strong** will be attributed to those with no WEAK ratings and at least two STRONG ratings;

**Moderate** will be given to those with one WEAK rating or fewer than two STRONG ratings;

**Weak** will be attributed to those with two or more WEAK ratings.

If four ratings have been given;

**Strong** will be attributed to those with no WEAK ratings and at least two STRONG ratings;

**Moderate** will be given to those with one WEAK rating or fewer than two STRONG ratings;

**Weak** will be attributed to those with two or more WEAK ratings.

The final decision of both reviewers will be: **strong, moderate, or weak**.

Results Summary

The 69 included studies used experimental and observational study designs. In terms of representativeness of the wider population, 64 of the studies were rated as ‘weak’ due to convenience sampling, with 3 studies rated as ‘moderate’ and 1 study rated as ‘strong’. We were able to rate 17 studies for representativeness relating to withdrawals and drop-outs: 4 studies were rated as ‘strong’, 10 studies were rated as ‘moderate’ and 3 received a ‘weak’ rating. 63 studies received a ‘moderate’ rating for confounding, while 5 studies were at a low risk of confounding and 1 was at high risk. 51 studies scored ‘strong’ on data collection, 16 scored ‘moderate’ and 2 studies scored ‘weak’. All 69 studies received a ‘strong’ score with regards to data analysis. 22 studies had a ‘moderate’ score in reporting quality, while 45 studies received ‘strong’ ratings and 2 received ‘weak’ ratings (see Web Appendix 3).

*Figure 1. Distribution of study quality scores across ratings, per Table 1*

**Web Appendix 4. Review of time-discounting measurement differences**

We identified heterogeneous methods used to measure time-discounting, based on i) how the survey is administered, ii) how the discount rate is estimated iii) and the nature of the rewards linked to the hypothetical choices.

All surveys were administered as either a paper or online questionnaire or asking the subject to make choices as options change incrementally on a computer. In both approaches, individuals state whether they would prefer a smaller reward now or a larger reward at some specified later time. This follows Kirby et al.’s ‘Monetary Choice Questionnaire’ method, in which choices vary by the size of reward and how long they must wait for the reward (3). The number of questions ranged from 2 (for example, Reimers et al. 2009) to 27 (for example, Audrain-McGovern et al. 2009). These responses are used to estimate discount rates by ascertaining an individual’s ‘indifference point’, where respondents switch from preferring a delayed over immediate reward, or vice versa. In other words, at this indifferent point they ascribe equal value to the immediate versus delayed (but larger) reward (4,5). Based on this first step, the researchers then estimate discount rates, either by fitting a hyperbolic model which assumes a ‘dynamic inconsistency’ (6,7) or by calculating the ‘Area Under the Curve’ of the estimated discount rates.

The final difference is the reward type. In 7 studies the rewards were ‘real’, such as cash payments or cigarettes, and all were honoured at the end of the survey (8–14). In contrast, in 41 studies the choices were hypothetical, including hypothetical money (55 studies), cigarettes (13 studies), health (5 studies) and food (2 studies). Estimation of non-monetary rewards followed a similar procedure to money: respondents chose between a specified number of cigarettes, better health, or a given food item immediately or a larger, delayed reward of the same type. An intermediate approach, used in 14 studies, is encourage respondents to treat their responses as real by rolling a dice or selecting one response at random at the end of the survey and honouring it, which are called ‘quasi-real’ rewards (11,15–34).

The contemporaneous association between time-discounting and smoking was replicated using a variety of discounting measures. 40 of these studies measured discount rates using hypothetical monetary reward measures, of which 34 (81.8%) reported significantly higher discount rates among smokers or smokers with higher consumption levels. All five studies of smoking initiation used hypothetical monetary discounting rewards and found that higher discounting predicted future smoking. 14 studies measured discount rates using ‘quasi-real’ rewards; 10 (71.4.5%) reported significantly higher discount rates among smokers (11,15–24,26,29,30,32). Of 7 studies using real monetary rewards, 4 (57.1%) reported higher discount rates among smokers (10,12,13,35). Among the 6 studies measuring discounting of hypothetical cigarette rewards, 5 (83.3%) reported higher discount rates among smokers (36–43) . Finally, among the 6 studies measuring hypothetical health rewards, four (66.6%) reported higher discount rates in smokers (36,39,40,44–46).

**Bibliography**

1. Moher D, Liberati A, Tetzlaff J, Altman DG, The PRISMA Group. Preferred reporting items for systematic reviews and meta-analyses: The PRISMA statement. Annals of internal medicine. 2009. p. 264–9.

2. Thomas BH, Ciliska D, Dobbins M, Micucci S. A process for systematically reviewing the literature: providing the research evidence for public health nursing interventions. Worldviews Evidence-Based Nurs. 2004;1(3):176–84.

3. Kirby KN, Petry NM, Bickel WK. Heroin addicts have higher discount rates for delayed rewards than non-drug-using controls. Journal of experimental psychology. General. 1999.

4. Green L, Myerson J, Ostaszewski P. Amount of reward has opposite effects on the discounting of delayed and probabilistic outcomes. J Exp Psychol Learn Mem Cogn. 1999;25(2):418–27.

5. Myerson J, Green L, Warusawitharana M. Area under the curve as a measure of discounting. J Exp Anal Behav. 2001;76(2):235–43.

6. Kahneman D, Knetsch JL, Thaler RH. Anomalies: The Endowment Effect, Loss Aversion, and Status Quo Bias. Journal of Economic Perspectives. 1991. p. 193–206.

7. Kahneman D, Tversky A. Prospect Theory: An Analysis of Decision under Risk. Econometrica. 1979;47(2):263–91.

8. Sheffer C, MacKillop J, McGeary J, Landes R, Carter L, Yi R, et al. Delay discounting, locus of control, and cognitive impulsiveness independently predict tobacco dependence treatment outcomes in a highly dependent, lower socioeconomic group of smokers. Am J Addict. 2012;21(3):221–32.

9. Anokhin AP, Golosheykin S, Grant JD, Heath AC. Heritability of delay discounting in adolescence: A longitudinal twin study. Behav Genet. 2011;41(2):175–83.

10. Reynolds B. The Experiential Discounting Task is sensitive to cigarette-smoking status and correlates with a measure of delay discounting. Behavioural pharmacology. 2006.

11. Reynolds B, Richards JB, Horn K, Karraker K. Delay discounting and probability discounting as related to cigarette smoking status in adults. Behav Processes. 2004;65(1):35–42.

12. Fields S, Collins C, Leraas K, Reynolds B. Dimensions of impulsive behavior in adolescent smokers and nonsmokers. Exp Clin Psychopharmacol. 2009;17(5):302–11.

13. Melanko S, Leraas K, Collins C, Fields S, Reynolds B. Characteristics of psychopathy in adolescent nonsmokers and smokers: Relations to delay discounting and self reported impulsivity. Exp Clin Psychopharmacol. 2009;17(4):258–65.

14. Yi R, Landes RD. Temporal and probability discounting by cigarette smokers following acute smoking abstinence. Nicotine Tob Res. 2012;14(5):547–58.

15. Fields S, Leraas K, Collins C, Reynolds B. Delay discounting as a mediator of the relationship between perceived stress and cigarette smoking status in adolescents. Behav Pharmacol. 2009;20(5-6):455–60.

16. Reynolds B, Fields S. Delay discounting by adolescents experimenting with cigarette smoking. Addiction. 2012;107(2):417–24.

17. Imhoff S, Harris M, Weiser J, Reynolds B. Delay discounting by depressed and non-depressed adolescent smokers and non-smokers. Drug Alcohol Depend. 2014;135(1):152–5.

18. Mitchell SH, Wilson VB. Differences in delay discounting between smokers and nonsmokers remain when both rewards are delayed. Psychopharmacology (Berl). 2012;219(2):549–62.

19. Lawyer SR, Schoepflin F, Green R, Jenks C. Discounting of hypothetical and potentially real outcomes in nicotine-dependent and nondependent samples. Exp Clin Psychopharmacol. 2011;19(4):263–74.

20. Chabris CF, Laibson D, Morris CL, Schuldt JP, Taubinsky D. Individual laboratory-measured discount rates predict field behavior. J Risk Uncertain. 2008;37(2-3):237–69.

21. Reynolds B, Patak M, Shroff P. Adolescent smokers rate delayed rewards as less certain than adolescent nonsmokers. Drug Alcohol Depend. 2007;90(2-3):301–3.

22. Perkins KA, Lerman C, Coddington SB, Jetton C, Karelitz JL, Scott JA, et al. Initial nicotine sensitivity in humans as a function of impulsivity. Psychopharmacology (Berl). 2008;200(4):529–44.

23. Conell-Price L, Jamison J. Predicting health behaviors with economic preferences & locus of control. J Behav Exp Econ. Elsevier Ltd.; 2015;54:1–9.

24. Heyman GM, Gibb SP. Delay discounting in college cigarette chippers. Behav Pharmacol. 2006;17(8):669–79.

25. Mitchell SH. Effects of short-term nicotine deprivation on decision-making: delay, uncertainty and effort discounting. Nicotine Tob Res. 2004;6(5):819–28.

26. Clewett D, Luo S, Hsu E, Ainslie G, Mather M, Monterosso J. Increased functional coupling between the left fronto-parietal network and anterior insula predicts steeper delay discounting in smokers. Hum Brain Mapp. 2014;35(8):3774–87.

27. Roewer I, Wiehler A, Peters J. Nicotine deprivation, temporal discounting and choice consistency in heavy smokers. J Exp Anal Behav. 2015;103(1):62–76.

28. Yamane S, Yoneda H, Takahashi T, Kamijo Y, Komori Y, Hiruma F, et al. Smokers, smoking deprivation, and time discounting. J Socio Econ. 2013;45:47–56.

29. Harrison GW, Lau MI, Rutström EE. Individual discount rates and smoking: Evidence from a field experiment in Denmark. J Health Econ. Elsevier B.V.; 2010;29(5):708–17.

30. Fields SA, Sabet M, Peal A, Reynolds B. Relationship between weight status and delay discounting in a sample of adolescent cigarette smokers. Behav Pharmacol. 2011;22(3):266–8.

31. MacKillop J, Amlung MT, Wier LM, David SP, Ray LA, Bickel WK, et al. The neuroeconomics of nicotine dependence: A preliminary functional magnetic resonance imaging study of delay discounting of monetary and cigarette rewards in smokers. Psychiatry Res - Neuroimaging. 2012;202(1):20–9.

32. Lewis R, Harris M, Slone S a., Shelton BJ, Reynolds B. Delay discounting and self-reported impulsivity in adolescent smokers and nonsmokers living in rural Appalachia. Am J Addict. 2015;

33. Harris M, Penfold RB, Hawkins A, Maccombs J, Wallace B, Reynolds B. Dimensions of impulsive behavior and treatment outcomes for adolescent smokers. Exp Clin Psychopharmacol. 2014;22(1):57–64.

34. Reynolds B. Do high rates of cigarette consumption increase delay discounting?: A cross-sectional comparison of adolescent smokers and young-adult smokers and nonsmokers. Behav Processes. 2004;67(3):545–9.

35. Reynolds B, Karraker K, Horn K, Richards JB. Delay and probability discounting as related to different stages of adolescent smoking and non-smoking. Behav Processes. 2003;64(3):333–44.

36. Baker F, Johnson MW, Bickel WK. Delay discounting in current and never-before cigarette smokers: similarities and differences across commodity, sign, and magnitude. J Abnorm Psychol. 2003;112(3):382–92.

37. Bickel WK, Odum AL, Madden GJ. Impulsivity and cigarette smoking: Delay discounting in current, never, and ex-smokers. Psychopharmacology (Berl). 1999;146(4):447–54.

38. Friedel JE, DeHart WB, Madden GJ, Odum AL. Impulsivity and cigarette smoking: discounting of monetary and consumable outcomes in current and non-smokers. Psychopharmacology (Berl). 2014;231(23):4517–26.

39. Poltavski D V, Weatherly JN. Delay and probability discounting of multiple commodities in smokers and never-smokers using multiple-choice tasks. Behav Pharmacol. 2013;24(8):659–67.

40. Johnson MW, Bickel WK, Baker F. Moderate drug use and delay discounting: a comparison of heavy, light, and never smokers. Exp Clin Psychopharmacol. 2007;15(2):187–94.

41. Jones BA, Landes RD, Yi R, Bickel WK. Temporal horizon: Modulation by smoking status and gender. Drug Alcohol Depend. 2009;104(SUPPL. 1).

42. Odum AL, Baumann AAL. Cigarette smokers show steeper discounting of both food and cigarettes than money. Drug and Alcohol Dependence. 2007. p. 293–6.

43. Wilson AG, Franck CT, Terry Mueller E, Landes RD, Kowal BP, Yi R, et al. Predictors of delay discounting among smokers: Education level and a Utility Measure of Cigarette Reinforcement Efficacy are better predictors than demographics, smoking characteristics, executive functioning, impulsivity, or time perception. Addict Behav. Elsevier Ltd; 2015;45:124–33.

44. Odum AL, Madden GJ, Bickel WK. Discounting of delayed health gains and losses by current, never- and ex-smokers of cigarettes. Nicotine Tob Res. 2002;4(3):295–303.

45. Cairns J. Developments in discounting: With special reference to future health events. Resour Energy Econ. 2006;28(3):282–97.

46. Khwaja A, Silverman D, Sloan F. Time preference, time discounting, and smoking decisions. J Health Econ. 2007;26(5):927–49.
